# Quantifying Information via Shannon Entropy in Spatially Structured Optical Beams

**DOI:** 10.34133/2021/9780760

**Published:** 2021-11-11

**Authors:** Maria Solyanik-Gorgone, Jiachi Ye, Mario Miscuglio, Andrei Afanasev, Alan E. Willner, Volker J. Sorger

**Affiliations:** ^1^Department of Electrical and Computer Engineering, The George Washington University, Washington, DC 20052, USA; ^2^Department of Physics, The George Washington University, Washington, DC 20052, USA; ^3^Department of Electrical Engineering at University of Southern California, Los Angeles, California 90089, USA

## Abstract

While information is ubiquitously generated, shared, and analyzed in a modern-day life, there is still some controversy around the ways to assess the amount and quality of information inside a noisy optical channel. A number of theoretical approaches based on, e.g., conditional Shannon entropy and Fisher information have been developed, along with some experimental validations. Some of these approaches are limited to a certain alphabet, while others tend to fall short when considering optical beams with a nontrivial structure, such as Hermite-Gauss, Laguerre-Gauss, and other modes with a nontrivial structure. Here, we propose a new definition of the classical Shannon information via the Wigner distribution function, while respecting the Heisenberg inequality. Following this definition, we calculate the amount of information in Gaussian, Hermite-Gaussian, and Laguerre-Gaussian laser modes in juxtaposition and experimentally validate it by reconstruction of the Wigner distribution function from the intensity distribution of structured laser beams. We experimentally demonstrate the technique that allows to infer field structure of the laser beams in singular optics to assess the amount of contained information. Given the generality, this approach of defining information via analyzing the beam complexity is applicable to laser modes of any topology that can be described by well-behaved functions. Classical Shannon information, defined in this way, is detached from a particular alphabet, i.e., communication scheme, and scales with the structural complexity of the system. Such a synergy between the Wigner distribution function encompassing the information in both real and reciprocal space and information being a measure of disorder can contribute into future coherent detection algorithms and remote sensing.

## 1. Introduction

An electromagnetic field is a fundamental physical carrier of information. It is capable of reliably transmitting a modulated signal and collecting information about the propagation channel itself. With relevance to this IT-driven age, the two longstanding goals in information processing are (i) achieving higher channel capacity (i.e., throughput) and (ii) (pre)processing of collected information. However, the fundamental challenge of rigorous qualification and quantification of information in EM waves still remains a topic of debate including both physical and even semi-philosophical notions. Information theory stands out from most of other approaches in physics. Being a higher level of abstraction, it focuses on a configuration of a system under consideration in the context of its prehistory, similarly to thermodynamics, without attachment to a particular class of objects under study in its axiomatics. Out of the broad scope of studies where information theory has a potential to contribute, at this point, we exemplary explore its applications to electrical engineering and signal processing.

One way of defining information is associating it with the presence of *distinctive features*. For instance, human speech can carry up to 2^14^ distinctive sounds (due to 14 binary distinctive features, e.g., [1]), only a small subset of which is realized in any particular known language. The amount of information a human can transmit per unit sentence containing a fixed number of words is indirectly correlated with the amount of distinctive features the language can handle (i.e., language capacity). Translating this into optics, it has been understood that a monochromatic plane-wave photon in free space can carry a rather limited amount of distinctive features (i.e., polarization and wavelength), providing up to one bit of information per photon. Naturally, if one generates photons with up to one bit of information, one is also able to detect only 1-bit information per unit carrier. To use an analogy, consider “a marine biologist casting a fishing net with two inches wide meshes for exploring the life on the ocean, naturally one should not be surprised finding only sea-creature larger than two inches long” [2]. This stumbling block has been shifted with the seminal work by Allen et al. [3] where it was experimentally confirmed that laser beams are capable of carrying a well-defined orbital angular momentum (OAM). An ability of such laser modes to carry theoretically unbounded amount of information per photon, e.g., [4], dramatically expands the EM-field's “language capacity.”

There are several approaches to assess a signal's information capacity developed in modern information theory, e.g., [5, 6]. In many cases, information is defined with respect to a particular alphabet, giving up the generality offered by statistics in the foundation of information theory. Several groups used conditional information approach to quantify the signal capacity [7]. Here, we introduce the concept of expressing information as a measure of structure in a physical system by applying the Shannon information theory to singular optical beams. We discuss how the Wigner distribution function (WDF) can be taken as a corresponding probability distribution function accounting for partial *quantumness* of a shaped photon source. The important synergy between a comprehensive description of physical systems in their phase space, delivered by the WDF, and the generalized axiomatics of the information theory has the potential to conduce a cumulative approach to high-information-density telecommunications and adaptive signal processing techniques. We validate this theoretical framework by experimentally showing how increased structural complexity of wavefront-shaped optical beams, such as Hermite-Gauss (HG) modes and optical vortices [8, 9], can be analyzed using wavefront sensors.

## 2. Results

### 2.1. The WDF and Classical Information in Optics

The WDF belongs to the generalized Cohen's class of dual-domain distributions. It is simultaneously the most complete analytical description of an optical beam, and an observable that can be experimentally measured. It provides access to the spatial beam profile and its Fourier transform. The WDF in one spatial dimension can be defined as

(1) $$\begin{array}{c} W\left(x,k_{x}\right)=\frac{1}{2\pi }\int dy\;u\left(x+\frac{y}{2}\right)u^{*}\left(x-\frac{y}{2}\right)e^{-ik_{x}y}, \end{array}$$

where *x* is the variable in the coordinate space and *k* is the corresponding coordinate in reciprocal space. The following integrals have a probabilistic interpretation:

(2) $$\begin{array}{c} {\left|u\left(k\right)\right|}^{2}=\int dxW\left(x,k\right),\\ {\left|u\left(x\right)\right|}^{2}=\int dk\;W\left(x,k\right),\\ U_{\text{tot}}=\int dxdk\;W\left(x,k\right), \end{array}$$

where |*u*(*k*)|^2^ is the momentum distribution, |*u*(*x*)|^2^ is the intensity distribution, and *U*_tot_ is the total energy of the incoming signal. For a fully coherent light source, the WDF's Fourier-transformed function Γ(*x*) = *u*(*x* + *a*)*u*^∗^(*x* − *a*) is known as the mutual intensity used in wavefront sensing for turbulence analysis and adaptive detection techniques. For brevity, a list of useful optical properties of the WDF can be found elsewhere, e.g., [10, 11].

**Figure 1:**
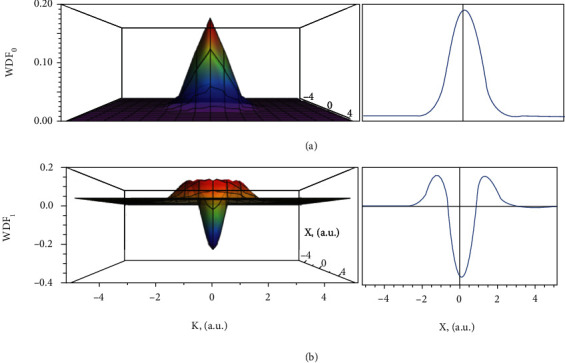
The Wigner distribution functions (WDFs) of one-dimensional (1D) Hermite-Gauss (HG) mode of zero-order HG_0_ (Gaussian) and first-order HG_1_ as functions of *κ*_*x*_-wave vector (5), and $\bar{x}$-coordinate in the beam's transverse plane. One can see that in the case of the Gaussian mode, the WDF is positive, while for the first-order HG mode, there is a negative contribution in the near-zero region of the phase space.

Now, let us consider a Gaussian beam, expressed in the following form [8]:

(3) $$\begin{array}{c} u_{G}\left(\rho \right)=\frac{A}{\text{w}}e^{-\left(\rho^{2}/{\text{w}}^{2}\right)}\;e^{i\tilde{k}\rho^{2}/2R}, \end{array}$$

where *ρ* > 0 is the position vector in the beam profile $\rho =\sqrt{x^{2}+y^{2}}$, w = w(*z*) is a beam waist, *R* = *R*(*z*) is the radius of curvature of the beam's wavefront, and *A* is the normalization constant. The wave-number $\tilde{k}=\left\{{\tilde{k}}_{x},{\tilde{k}}_{y},{\tilde{k}}_{z}\right\}$ is distinctive from the Fourier transform parameter *k* = {*k*_*x*_, *k*_*y*_, *k*_*z*_} in (1). The corresponding 1D WDF is [12]

(4) $$\begin{array}{c} W^{\left(G\right)}\left(\tilde{x},\kappa_{x}\right)=\frac{A}{\sqrt{2\pi }}e^{-{\tilde{x}}^{2}-\kappa_{x}^{2}}, \end{array}$$

which is plotted in position-momentum space {*x*, *k*_*x*_} in Figure 1(a). Here, $\bar{x}=\sqrt{2}x/\text{w}$ and *κ*_*x*_ is the redefined momentum:

(5) $$\begin{array}{c} \kappa_{x}=\frac{\text{w}}{\sqrt{2}}\left(k_{x}-\frac{{\tilde{k}}_{x}\;x}{R}\right). \end{array}$$

This Wigner distribution is properly normalized, delivering the beam intensity distribution when integrated over the entire momentum space.

Classical Shannon information, see [13], represents the amount of structure in the corresponding system. Its analog for an optical mode, characterised by its WDF, is

(6) $$\begin{array}{c} S=-\iint_{R^{2}}dr\;dk\;W\left(r,k\right)\cdot \ln\left[W\left(r,k\right)\right], \end{array}$$

where the WDF is a scalar function of position **r** and momentum **k** vectors. Similar definitions of information applied to characterizing optical fields, while having been suggested in the field of optics earlier, e.g., [12, 14], have not been explicitly applied to topological optical beams in the way introduced here, to the best of the authors' knowledge. The main problem with this definition, Equation (6), is that the WDF is not a positive-semidefinite function and, hence, does not represent a proper distribution. Negativity of the WDF can be interpreted as a marker of a phase-space interference and nonclassicality [15, 16]. However, in quantum description, interference is associated with a local violation of Heisenberg inequality, e.g., [17]. For these reasons, we seek a definition that would comply with the quantum nature of light.

Here, we propose to replace the regular product in the definition of information (6) with the Groenewold associative product [18].

(7) $$\begin{array}{c} ⋆=e^{i\hbar \left(\overset{⟵}{\partial_{x}}\overset{⟶}{\partial_{p}}-\overset{⟵}{\partial_{p}}\overset{⟶}{\partial_{x}}\right)/2}, \end{array}$$

and hereby call it Shannon-Groenewold information:

(8) $$\begin{array}{c} \tilde{S}=-\frac{1}{{\left(\pi \hbar \right)}^{2}}\iint_{{\mathbb{R}}^{2}}dr\;dk\;W\left(r,k\right)⋆\ln\left[W\left(r,k\right)\right], \end{array}$$

where *W*(**r**, *k*) is the four-dimensional (4D) WDF (see [19]). A manifestation of this definition is the Weyl quantization of a classical observable in phase space [20]. It respects the Heisenberg uncertainty principle and is normalized to the phase space volume, which is one of the main advantages of this definition of information over Shannon information Equation (6), defined via the WDF. As an entropic observable, it measures the structure present in the optical field analogous to [21], as opposed to the earlier attempts to utilize the conditional information [7], that is, by design, less fundamental due to being alphabet specific. In the context of Information Theory, it may be interpreted as a measure of the amount of *classical* information that can be extracted from the laser mode if all the quantum uncertainty is removed by an appropriate experiment.

Using the Shannon-Groenewold information (8), we obtain the following expression for the information in the Gaussian beam:

(9) $$\begin{array}{c} {\tilde{S}}_{1\text{D}}≃-U_{\text{tot}}\left[\ln\left(\frac{U_{\text{tot}}}{\pi }\right)-1\right], \end{array}$$

(10) $$\begin{array}{c} {\tilde{S}}_{2\text{D}}≃-U_{\text{tot}}^{2}\left[\ln\left(\frac{U_{\text{tot}}^{2}}{\pi^{2}}\right)-2\right], \end{array}$$

where $U_{\text{tot}}=A\sqrt{\pi /2}$ is the total EM energy of the beam per spatial degree of freedom. We assume that the radius of curvature of the beam wavefront is always larger than the physical dimensions of the beam spot size: *R* ≫ {*x*, *y*}. It is important to note that for the Gaussian mode, both definitions (6) and (8) produce the same result. This is what one would expect since the WDF of pure Gaussian sources is positive-semidefinite and can be interpreted as a classical proper distribution function [22]. The fact that information is energy-dependent comes as no surprise if one remembers the important synergy between information and entropy in classical Shannon theory of information (see [13]). Even though entropy in information theory and thermodynamic entropy are not exactly identical, one would expect similar behavior when it comes to the fundamental laws of physics [23, 24]. In this context, total energy of a laser mode is in equivalence with total internal energy of a thermodynamic system.

### 2.2. Higher-Order Modes and Shannon-Groenewold Information

The HG mode is a higher-order solution of the Gaussian family of beam-like solutions of the paraxial equation. It carries a nontrivial beam geometry with singularities, stable on propagation [25] due to being topologically protected. Hence, it is only logical to expect that the amount of information for this family of modes is higher than for the zero-order Gauss modes ${\tilde{S}}_{G}≲{\tilde{S}}_{HG}$ [26]. Let us investigate this statement next.

The general solution of the 1D paraxial wave equation in cylindrical coordinates is given by

(11) $$\begin{array}{c} u_{HG}\left(x,z\right)=\sqrt{\frac{A}{\text{w}}}H_{m}\left(\frac{\sqrt{2}x}{\text{w}}\right)e^{-\left(x^{2}/{\text{w}}^{2}\right)}e^{i{\tilde{k}}_{x}x^{2}/2R}. \end{array}$$

The normalization is consistent with the one in Equation (3). The WDF for this mode is known, e.g., [27]. In our case, to keep the normalization consistent, we obtain

(12) $$\begin{array}{c} W_{m}^{\left(HG\right)}\left(x,k_{x}\right)=\frac{A\sqrt{\pi }}{{\left(2\pi \right)}^{3/2}}e^{-{\bar{x}}^{2}}\int_{-\infty }^{\infty }d\bar{y}\;e^{-i\kappa_{x}\;\tilde{y}-\left({\bar{y}}^{2}/4\right)}H_{m}\left(\bar{x}+\frac{\bar{y}}{2}\right)H_{m}\left(\bar{x}-\frac{\bar{y}}{2}\right). \end{array}$$

As expected, for *m* = 0, the WDF of HG mode is exactly equal to the Gaussian WDF (Equation (4)) and so is the corresponding information in Equation (9). Then, the first two higher-order WDFs have “elegant” analytical expressions:

(13) $$\begin{array}{c} W_{1}^{\left(HG\right)}\left(x,k_{x}\right)=\frac{2A}{\sqrt{2\pi }}e^{-{\bar{x}}^{2}-\kappa_{x}^{2}}H_{1}\left({\bar{x}}^{2}+\kappa_{x}^{2}-\frac{1}{2}\right), \end{array}$$

(14) $$\begin{array}{c} W_{2}^{\left(HG\right)}\left(x,k_{x}\right)=\frac{4A}{\sqrt{2\pi }}e^{-{\bar{x}}^{2}-\kappa_{x}^{2}}H_{2}\left({\bar{x}}^{2}+\kappa_{x}^{2}-1\right). \end{array}$$

The WDF *W*_1_^(*HG*)^(*x*, *k*_*x*_) is plotted in Figure 1(b). One can see explicitly the negative contributions, in the 2nd-order mode in opposition to the positive-definite 0th-order Gauss beam. In a similar manner, one can work out higher-order modes using the integral form in Equation (12).

Next, we explore these WDF expressions further. We find that these WDFs take negative values in the central region of the phase space (Figure 1). The fundamental statement of classical information theory that “the more we know about a system's parameter space, the less is its uncertainty” is inevitably broken in the quantum context when considering correlated (conjugate) variables, such as position *x* and momentum *p*. If such “quantumness” is present in the corresponding PDF, it pushes the distribution into the negative domain [16] (Figure 1(b)). By respecting Weyl-Wigner quantization in the definition of information (8), we work around the WDF not being a well-defined PDF from the statistics point of view. Groenewold's product, introduced instead of conventional multiplication, enforces the negative regions to be integrated out, in compliance with the Heisenberg uncertainty relation. These lead to real and positive-valued, monotonously increasing with energy information:

(15) $$\begin{array}{c} {\tilde{S}}_{1\left(1\text{D}\right)}^{\left(HG\right)}≃-2U_{\text{tot}}\left[\ln\left(\frac{2U_{\text{tot}}}{\pi }\right)-3\right], \end{array}$$

(16) $$\begin{array}{c} {\tilde{S}}_{2\left(1\text{D}\right)}^{\left(HG\right)}≃-8U_{\text{tot}}\left[\ln\left(\frac{4U_{\text{tot}}}{\pi }\right)-5\right]. \end{array}$$

As HG modes form a complete orthonormal set, they can be used as an expansion basis [28]. Hence, this approach can be straightforwardly applied to OAM modes, such as Laguerre-Gauss (LG), e.g., [8]. Let us express the LG mode as follows:

(17) $$\begin{array}{c} u_{LG}\left(\rho ,z\right)=\frac{A}{w}{\left(\frac{\sqrt{2}\rho }{w}\right)}^{\left|\ell \right|}L_{p}^{\left|\ell \right|}\left(\frac{2\rho^{2}}{w^{2}}\right)e^{-\left(\rho^{2}/w^{2}\right)}e^{ik\rho^{2}/2R}e^{i\ell \phi }, \end{array}$$

where *ℓ* is the vorticity of the twisted mode and *ϕ* is the azimuthal angle in cylindrical coordinates, where the remaining parameters follow the definitions of Gauss (3) and HG (11) modes. Supplying the results from (12), we can express LG modes in terms of HG modes with a straightforward calculation; for instance, for the WDF of the 2D LG mode with *p* = 0 and *ℓ* = 1,

(18) $$\begin{array}{c} W_{1\left(2\text{D}\right)}^{\left(\text{LG}\right)}\left(\bar{\rho };\kappa \right)=\frac{A^{2}}{2\pi }e^{-{\bar{\rho }}^{2}-\kappa^{2}}\left({\bar{\rho }}^{2}+\kappa^{2}-1\right), \end{array}$$

where *p* and *ℓ* are correspondingly the order and degree numbers of the generalized Laguerre polynomial *L*_*p*_^*ℓ*^(·); $\bar{\rho }=\sqrt{{\bar{x}}^{2}+{\bar{y}}^{2}}$. The corresponding entropy can be calculated in a similar manner:

(19) $$\begin{array}{c} {\tilde{S}}_{1\left(2D\right)}^{\left(\text{LG}\right)}≃-2U_{\text{tot}}^{2}\left(\ln\left(\frac{U_{\text{tot}}^{2}}{\pi^{2}}\right)-4\right). \end{array}$$

**Figure 2:**
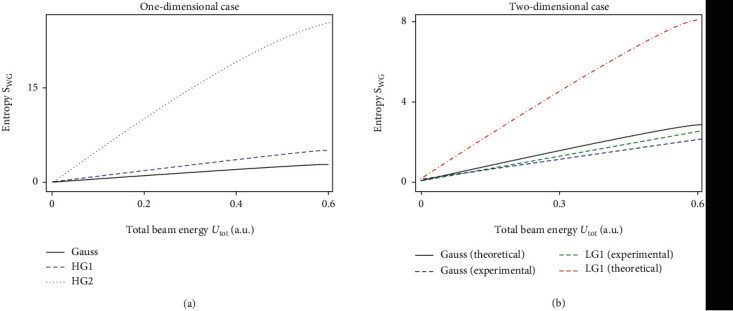
The Shannon-Groenewold information *S*_WG_ as a function of total energy *U*_tot_ (8) for one-dimensional (a) HG_0_/Gauss mode (black), HG_1_ (dashed-yellow), and HG_2_ (dotted-green), and two-dimensional (b) theoretically predicted (black) and experimental (dashed-blue) Gauss mode, experimentally measured (long-dashed-green) and theoretically predicted (dot-dashed-red) LG_1_ with the corresponding error bands resulting from the errors on fit parameters shown in light-red; see Section 3 for details. One notices the overall tendency for the amount of information to increase with the growth of the overall complexity of the corresponding optical signal. Also, the drop in the amount of information inferred from the experiment as compared to the theoretical curve is attributed to the SLM's beam conversion efficiency and expected information loss during the propagation in free space.

The classical assessment of the amount of order in the optical mode is clearly increasing with the increasing complexity of the beam profile (see Figure 2), as one would expect from general considerations. It is important to recall that in the context of physical meaning of Shannon unconditional information, the WDF is normalised to *U*_tot_—total energy. Consequently, constant *A* is bounded from above by $\sqrt{2/\pi }$.

The similarity sign in the expressions for information (structural complexity) of the considered above modes (9), (15), (16), and (19) is due to the presence of terms containing an odd logarithmic integral of the form:

(20) $$\begin{array}{c} \int_{0}^{\infty }dr\;r\;\ln\left(2r^{2}-1\right)I_{0}\left(\alpha r\right)⟶0, \end{array}$$

where *I*_0_ is a modified Bessel function of the first kind. Numerical estimations show a tendency for these terms to go to zero; however, mathematically rigorous study of their absolute convergence has not been performed.

**Figure 3:**
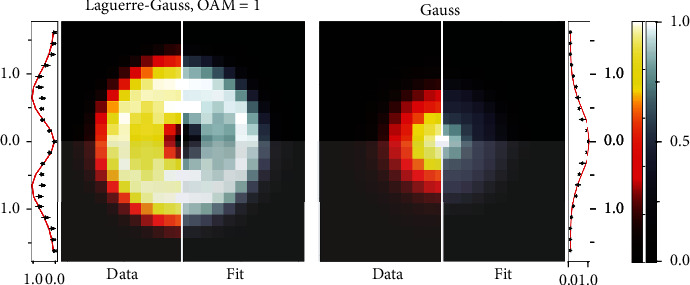
On the way to measuring the amount of information in optical beams. The intensity distributions of the EM beams are captured by a SHS. The algorithm for simulated data sets and the fits is based on Equations (21) and (22). The experimental data are averaged over 4 Laguerre-Gaussian LG_1_ samples (left) and 10 Gaussian samples (right) with the SHS's sampling rate of 18 fps (black-starred scatter plot) and over the four quadrants in the beam intensity profile. In 2D color maps, measurement data is shown in gray scale, simulation is depicted in orange-red scale, and shaded regions are symmetry-based extrapolations. The measurement fits are shown in solid-red curves with the Gaussian model (right) using the WDF in Equation (4), and Laguerre-Gauss (LG) model (left) in Equation (18). The measured data are shown as black stars with the corresponding error bars. All the presented plots depict normalized intensity *I* ∈ [0, 1].

With this theoretical framework, we performed an experiment to explore the possibility of obtaining the amount of information by measuring the structure in optical laser beams of various topology. Provided the wavefront reading was obtained from an off-the-shelf Shack-Hartmann sensor, we assessed the amount of information in several fundamental laser modes (see Figure 3).

## 3. Materials and Methods

Among the tools of adaptive optics, Shack-Hartman sensors (SHS) [29] occupy a unique place as a fast, affordable, and compact off-the-shelf tool for simultaneous intensity and angular distribution measurements. Advanced techniques for SHS state tomography [30] and WDF reconstruction [31] have been suggested alongside with conventional aberration correction techniques. Measurements of the wavefront distortions in EM beams with nontrivial topology are also of interest for both communication and sensing purposes, e.g., [32].

**Figure 4:**
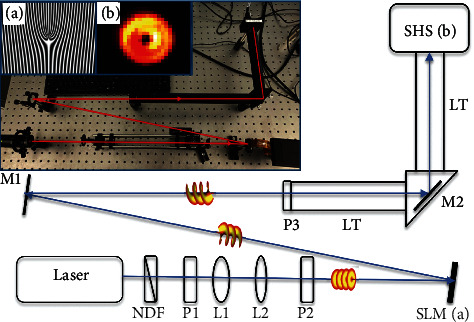
The schematic of the experimental setup, where a spatially filtered Gauss-like 635 nm, 4 mW (Thorlabs LDM635) laser beam is reflected off the spatial light modulator (SLM) onto the mirror M1, into the boxing of the Shack-Hartmann sensor (SHS) (Thorlabs: WFS20-7AR). The device is optimized to operate with ordinary Gauss-like signals. The SLM can be set to a mirror regime or to generate an orbital angular momentum (OAM) beam of Laguerre-Gauss-like profile. The OAM beam generation is accomplished by an SLM loaded with a computer-generated diffraction pattern with a fork dislocation (i). In that case, the measured intensity distribution of an OAM beam (ii) has a typical doughnut-like structure. L1 and L2 are the lenses of the beam expander; P1, P2, and P3 are the pinholes; M1 and M2 are the directing mirrors; and LT are the boxing elements of the SHS.

For the aim of this work, we use the SHS to reconstruct the WDF for the purpose of discriminating between the modes, assessing the beam quality and ultimately the amount of information in a beam. While a SHS is utilized generally to facilitate beam alignment and assess distortions within an optical channel by calculating the higher-order Zernike moments, here, we omit the SHS's wavefront data output and only consider the raw data of the intensity distribution in the superpixel array of the SHS's camera (Figure 4). We compare the results to the modelled intensity distribution based on the theoretical WDF calculation, whose approximation can be modelled as [31]

(21) $$\begin{array}{c} I\left(r\right)=\frac{1}{\lambda f}\overset{L,M}{\underset{\begin{array}{c} \ell =-L\\ m=-M \end{array}}{\sum }}\text{S}\text{WDF}\left[W_{b},W_{a}\right]\left(r_{\ell ,m}^{\prime },u_{\ell ,m}^{\prime }\right)\text{rect}\left(r_{\ell ,m}^{\prime }\right). \end{array}$$

The smooth WDF is defined as follows [33]:

(22) $$\begin{array}{c} \text{SWDF}\left[W_{b},W_{a}\right]\left(r_{\ell ,m}^{\prime },k_{\ell ,m}^{\prime }\right)=\iint d^{2}R\;d^{2}UW_{b}\left(R,U\right)W_{a}\left(R-r_{\ell ,m}^{\prime },U-u_{\ell ,m}^{\prime }\right), \end{array}$$

where the coordinate shift is defined as

(23) $$\begin{array}{c} r\prime_{\ell ,m}=\left\{R_{x}-\ell w,R_{y}-mw\right\},\\ u\prime_{\ell ,m}=\left\{U_{x}-\frac{x-\ell w}{\lambda f},U_{y}-\frac{y-mw}{\lambda f}\right\}. \end{array}$$

The functions *W*_*b*_ and *W*_*a*_ are the WDF of the incoming signal, e.g., (4), (13), (14) or (18), and the transmission function of a single lens aperture correspondingly. The parameters *f* (focal length) and *w*(width of a single lens in a lenslet array) are the parameters specific to the detector and define the angles in the local wavefront of the field.

Using this model, we first simulated the synthetic data sets for Gauss, HG, and LG modes. In the model, we considered the WDF *W*_*b*_ in the following general form:

(24) $$\begin{array}{c} W\left(\tilde{x},\kappa_{x}\right)=\frac{A}{\sqrt{2\pi }}e^{-{\tilde{x}}^{2}-\kappa_{x}^{2}}\;P\left({\tilde{x}}^{2}+k_{x}^{2}\right), \end{array}$$

where *P*(*α*) is the polynomial of *α*. We started by testing two cases, namely, a Gauss mode (4) and a LG of order 1 (18). The polynomial fit in Equation (24) was taken to be

(25) $$\begin{array}{c} P\left({\tilde{x}}^{2}+k_{x}^{2}\right)=a+b\left({\tilde{x}}^{2}+\kappa_{x}^{2}\right), \end{array}$$

with *a* and *b* being the fitting parameters (Figure 3). One can see that when *a* = const and *b* = 0, the fit corresponds to a Gaussian profile (4), and when *a*/*b* = 2, the model includes LG_1_-like distributions (18).

**Figure 5:**
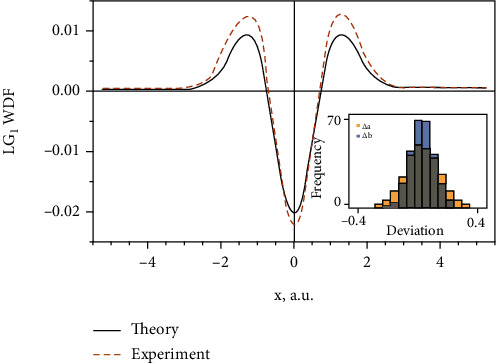
Comparing the experiment and the theoretical prediction for the WDF of the LG mode of order *ℓ* = 1: (a) the WDF of the ideal LG_1_ mode (solid black) and of the measured in an experiment laser beam (dashed yellow) with the corresponding error band; the frequency histogram of the deviation of the fitting parameters *a* (yellow) and *b* (blue) in the inset at the right-bottom, Equation (25), supplied to the model (21), and resulted from the fitting procedure: 1000 synthetic data sets have been generated and fitted with the model, mentioned above; for each run, the deviation between the supplied and fitted parameters has been calculated.

To assess the quality of the model, besides estimating the *χ*^2^ per each fit, we run a simulation with 1000 fits to synthetic data with Equation (21), obtaining a histogram of the deviation between the supplied fitting parameters and those from the best fit (Figure 5(b)). The resulting data show that the parameters are centered near the “true” values, showing the satisfactory quality of the fit.

The inferred fundamental amount of information carried in an experimentally measured photon beam appears to be lower than the theoretically predicted for the ideal LG_1_ mode (see Figure 2). The resulting entropy as a function of beam energy is extremely sensitive to the fit parameters *a* and *b* (25).

Since we did not consider the wavefront readings of the SHS, this model does not harvest the information stored in the reciprocal domain at this point, but rather infers it from the intensity distribution. This capability is to be explored and utilized in our future research. However, even at this conceptual level, the fit already can discriminate between the two modes. Hence, it provides an experimental estimate for the WDF (Figure 5(a)) supplied, however a “good” guess about the possible wavefront shape of the incoming signal. Due to intensity-only detection, the central region of the LG beam, generated in the experiment, is left out. Hence, the setup is inherently classical, in compliance with the definition of information, used here.

The quality of the fit, alongside with the accuracy of the numerical integration algorithm, also depends on the detector modelling scheme, (21) and (22), which in this case has been fairly generic. One of the crucial assumptions that has been made is the plane-wave approximation. This approximation, generally speaking, is too brave for the case of topological beams. Another unaccounted source of discrepancies is the optical cross-talk due to SHS's architecture that has been extensively discussed before on the level of mathematical modelling [31]. Hence, this model, thought already fruitful, has a great potential for improvement.

These results were used to assess the information stored in a physical channel and to compare them to theoretical curves (Figure 2). As for applications, the full 2D model can also provide information about the medium the beam interacted with that can be useful in remote sensing. Based on the results of Section 2.2, in the course of future research, we expect topological beams to outperform the modes with planar phase structure for two main reasons: (1) greater library of nontrivial signatures in the original beam profile; (2) reported robustness and self-healing properties of vortex modes.

## 4. Discussion

Interestingly, while information technologies have seen outstanding progress over the last century, which lead to the digital revolution and created flourishing businesses, the field of information theory has remained in a shade. We believe that a universal technique to assess both the quality and quantity of information in a received signal, if provided, could become a conceptually novel tool to physicists and engineers alike. The approach described in this work is by far not the first attempt, neither is it the most general. However, in this approach, classical information does not require an early choice of a communication scheme (i.e., alphabet). It is rather based on a fundamental assessment of an optical system's capability to carry information, based on its overall complexity. The WDF is uniquely used here as a probability density function for Shannon information in optics. The constraints of probability theory on the definition of information and of quantum mechanics on conjugate observables are satisfied working around the properties of the WDF—a *pseudo*-probability distribution. We foresee the relevance of this formalism in the context of recent developments for (i) free-space information-processing optics [34]; (ii) integrated photonics-based information processing [35] such as neural network-based accelerators [36] and photonic tensor cores [37]; (iii) adaptive sensing [38]; and (iv) analog optical and photonic processors [39–41]. As the data compression coefficient is naturally bounded by Shannon information, carried by the beam [42], this work indirectly points towards higher information capacity in beams with a nontrivial structure, like HG, LG, and Bessel-Gauss modes [43, 44].

Due to the WDF's relation to the EM-field correlation function, we foresee our approach to be extremely useful in adaptive optics. The reconstruction algorithm, when fully developed, has the potential to characterize the effects of decoherence in turbulent media, the 2D ambiguity function, and time-resolved frequency distribution, alongside with commonly available corrections for aberration, astigmatism, peak valley, and rms deformation provided by the SHS measurements. The WDF formalism uniquely gives access to such characteristics as mutual intensity of stochastic wave fields, which is of high importance when describing partially coherent sources.

In perspective, as the demand on high-speed data transfer and streaming grows exponentially, ADSL and fiber-to-home technologies are less and less likely to satisfy even an average consumer's data hunger, not to mention business and government agency calls. These, together with the recent advents in optical processing [34], micro- and nanofabrication [45], and OAM communications [46] put forward the mid-20th century's excitement around free-space communications in a new light. The new-generation free-space links will require coherent detection techniques to realise their potential to the fullest. For instance, the idea of using classical information as a quantifier for the amount of information processing that can be done on a given metasurface has already been implemented [47]. This approach elegantly gauges a specific pattern on the surface of a metastructure (lens) with the corresponding distribution of electromagnetic radiation in the far field [48]. With this progress, we believe that our approach may result in a better understanding of which types of measurements and device architectures are needed to efficiently mine information from a free-space link.

## Data Availability

The data used in this research have been acquired manually in the OPEN Lab facility, the George Washington University, and can be shared by request.

## Supplementary Materials

Supplementary Materials The authors provide supplementary information that offers details on some of the derivations and the theoretical approach presented in this paper.
