# Supplementary material for: Quantifying Information via Shannon Entropy in Spatially Structured Optical Beams
Source: Research (Wash D C). 2021 Nov 11;2021:9780760. doi: 10.34133/2021/9780760 (PMC8605402; doi:10.34133/2021/9780760)
Supplement: Supplementary Materials — The authors provide supplementary information that offers details on some of the derivations and the theoretical approach presented in this paper. [file 9780760.f1.pdf]

Quantifying information via Shannon entropy in spatially structured optical beams

Supplementary material

Here we provide some details on the computations and derivations that stand behind the formalism. Let's start with the Groenewold's product in its integral form is defined as:

$$f(x,p) \star g(x,p) = \int d(u,v,\omega,z) f(x+u,p+v) g(x+\omega,p+z) e^{\frac{2i}{\hbar}(uz-v\omega)} \quad (1)$$

In our calculations of information eqn. (10), $f(x,p)$ is the corresponding WDF and $g(x,p)$ is its logarithm. For instance, for Gauss beams in one-dimensional case one gets:

$$\tilde{S}_{1D}^{(G)} = -\frac{A}{\sqrt{2\pi}} \int d\tilde{x} d\tilde{\kappa}_x \, e^{-x^2-\kappa_x^2} \star \left[\ln\left(\frac{A}{\sqrt{2\pi}}\right) - \tilde{x}^2 - \kappa_x^2 \right] \quad (2)$$

which results into a six-fold integral. The Groenewold's product can be expressed as:

$$\begin{gathered} \frac{A}{\sqrt{2\pi}} e^{-x^2-\kappa_x^2} \star \left[\ln\left(\frac{A}{\sqrt{2\pi}}\right) - \tilde{x}^2 - \kappa_x^2 \right] = \\ \frac{A}{\sqrt{2\pi}} \int d(u,v,\omega,z) \, e^{-(x+u)^2-(\kappa_x+v)^2+\frac{2i}{\hbar}(uz-v\omega)} \times \\ \times \left(-(x+\omega)^2 - (\kappa_x+z)^2 + \ln\frac{A}{\sqrt{2\pi}} \right) = \\ \frac{A}{\sqrt{2\pi}} \pi^2 \hbar^2 e^{-\kappa_x^2-x^2} \left(\ln\frac{A}{\sqrt{2\pi}} - \kappa_x^2 - x^2 + \hbar^2(-1+\kappa_x^2+x^2) \right) \end{gathered}$$

One can check by straightforward calculation, that by substituting this expression into (29), one arrives to first equation in (11).

The higher-order modes employ the following integrals:

$$\int_{\mathbb{R}} d(u,v,w,z) e^{\frac{2i}{\hbar}(uz-vw)-u^2-v^2-2(xu+kv)} = \hbar^2\pi^2 \quad (3)$$

$$\int_{\mathbb{R}} d(u,v,w,z) u^\alpha v^\beta e^{\frac{2i}{\hbar}(uz-vw)-u^2-v^2-2(xu+kv)} = 0 \quad (4)$$

$$\begin{aligned} &\int_{\mathbb{R}} d(u,v,w,z) u^\alpha v^\beta e^{\frac{2i}{\hbar}(uz-vw)-u^2-v^2-2(xu+kv)} \times \\ &\times \ln[H_1\big((x+w)^2+(k+z)^2-1/2\big)] \xrightarrow[1/\hbar^2\to\infty]{} 0 \end{aligned} \quad (5)$$

where $\mathrm{Re}[\hbar^2] > 0$ is a positive constant, and $\alpha, \beta \in \mathbb{Z}^+$ are any two positive integers, excluding the case $\alpha = \beta = 0$. The calculus gets more technically involved, but remains the same procedure-wise.

We used the following expression to express Laguerre-Gauss modes in terms of Hermite-Gauss functions:

$$e^{i\ell\phi}\rho^{\ell}L_p^{\ell}(\rho^2) = \frac{(-1)^p}{2^{(2p+\ell)}p!}\sum_{r=0}^{p}\sum_{s=0}^{\ell} i^s \binom{p}{r}\binom{\ell}{s} H_{2r+\ell+s}(x)H_{2p-2r+s}(y) \tag{6}$$

where $\rho = \sqrt{x^2+y^2}$ is the unit-vector in cylindrical coordinate system.
